# Low-Grade Endometrial Stromal Sarcoma with Intravenous and Intracardiac Extension: A Multidisciplinary Approach

**DOI:** 10.1155/2016/3467849

**Published:** 2016-04-27

**Authors:** Wataru Kudaka, Hitoshi Inafuku, Yuko Iraha, Tomoko Nakamoto, Yusuke Taira, Rie Taira, Hisashi Kamiya, Maho Tsubakimoto, Yuichi Totsuka, Yukio Kuniyoshi, Tomoko Tamaki, Hajime Aoyama, Masanao Saio, Naoki Yoshimi, Yoichi Aoki

**Affiliations:** ^1^Department of Obstetrics and Gynecology, Graduate School of Medicine, University of the Ryukyus, 207 Uehara, Nishihara, Okinawa 903-0215, Japan; ^2^Department of Thoracic and Cardiovascular Surgery, Graduate School of Medicine, University of the Ryukyus, 207 Uehara, Nishihara, Okinawa 903-0215, Japan; ^3^Department of Radiology, Graduate School of Medicine, University of the Ryukyus, 207 Uehara, Nishihara, Okinawa 903-0215, Japan; ^4^Department of Pathology and Oncology, Graduate School of Medicine, University of the Ryukyus, 207 Uehara, Nishihara, Okinawa 903-0215, Japan

## Abstract

*Background*. A rare case of low-grade endometrial stromal sarcoma (LG-ESS) extending to inferior vena cava (IVC) and cardiac chambers.* Case Report*. A 40-year-old woman had IVC tumor, which was incidentally detected by abdominal ultrasonography during a routine medical checkup. CT scan revealed a tumor in IVC, right iliac and ovarian veins, which was derived from the uterus and extended into the right atrium and ventricle. The operation was performed, the heart and IVC were exposed, and cardiopulmonary bypass was initiated. A right atriotomy was performed, and the intracardiac mass was removed. Then the tumor in IVC and the right internal iliac vein were removed after longitudinal venotomies in the suprarenal and infrarenal vena cava, the right common iliac vein. Next the pelvis was explored. Tumors were found originating from the posterior wall of the uterus and continuing into both the right uterine and ovarian vein. The patient underwent total hysterectomy with bilateral salpingooophorectomy. Complete tumor resection was achieved. Histopathological analysis confirmed a diagnosis of LG-ESS. She showed no evidence of disease for 2 years and 3 months.* Conclusions*. Our case highlights the importance of a multidisciplinary approach in treating this rare cardiovascular pathological condition through preoperative assessment to final operation.

## 1. Introduction

Low-grade endometrial stromal sarcoma (LG-ESS) has indolent clinical progression with repeated local recurrence within 10 years up to 50% of cases. Late mortality because of lung metastases occurs in 15% of cases [1, 2]. ESS tends to spread throughout the lymph nodes and venous system but rarely involves large vessels or the heart. In general, the intracardiac extension of LG-ESS is rare and most cases derive from renal cell carcinoma, nephroblastoma, colon adenocarcinoma, melanoma, hepatocellular carcinoma, or bronchogenic carcinoma [3]. Only 22 cases of advanced LG-ESS have been reported in which the great vessels were invaded and a tumor of the inferior vena cava (IVC) formed [3–6]. However, earlier studies show that >50% of intravenous LG-ESS cases exhibit intracardiac extension [7].

Here, we report a rare case of LG-ESS extending to IVC and cardiac chambers, which was treated with a multidisciplinary approach. Written informed consent was obtained from the patient for the publication of her medical details.

## 2. Case Presentation

**Figure 1:**
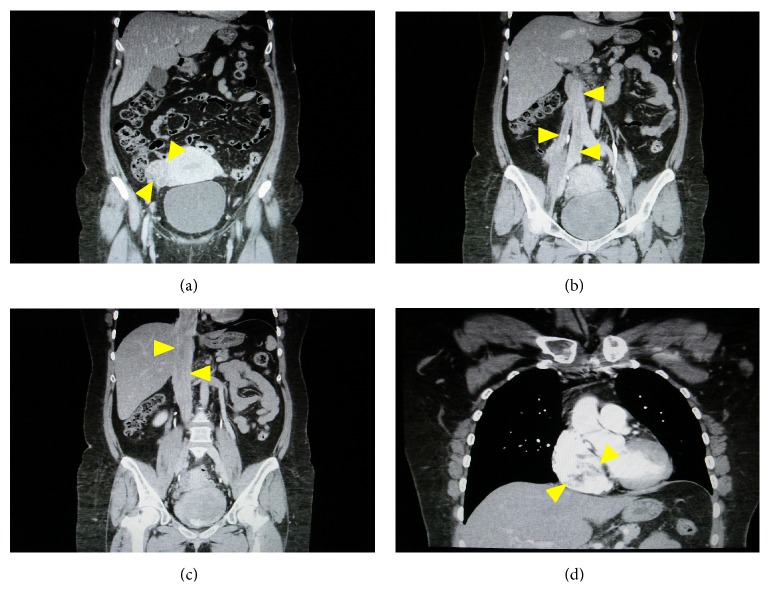
Computed tomogram of the chest, abdomen, and pelvis showed a large mass within the inferior vena cava, extending into the right atrium and ventricle. (a) Coronal image demonstrates large filling defect in the right parametrium. (b, c) Coronal image presents large filling defect in the inferior vena cava extending from both the right uterine vein and common iliac and the right ovarian vein to the inferior vena cava and the right atrium. (d) Coronal image demonstrates large filling defect in the right atrium.

**Figure 2:**
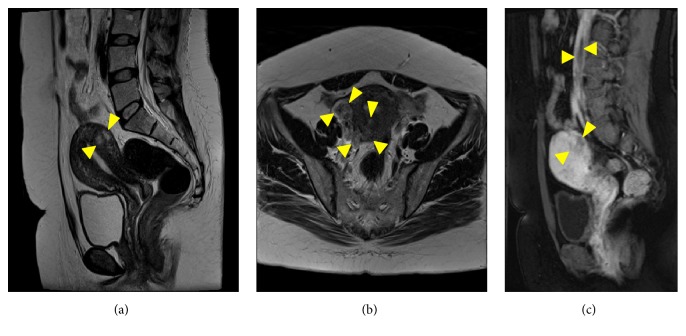
Magnetic resonance imaging (MRI): (a) on T2 weighted image, an irregular tumor is identified in the right posterior wall of the uterus, which exhibited heterogeneous high signal intensity (arrowheads); (b) the right uterine vein and ovarian vein are dilated, and low and high intensity masses are depicted in the veins and originated from the high intensity tumor in the posterior uterine wall (arrowheads); (c) sagittal view of dynamic contrast-enhanced MRI demonstrates large filling defect in the inferior vena cava with gradually increasing enhancement (upper arrowheads) and poor enhancement of tumor in the posterior uterine wall (lower arrowheads).

A 40-year-old woman had received regular follow-up for a fatty liver, and inferior vena cava (IVC) tumor, which was initially thought to be a blood thrombus, was incidentally detected by abdominal ultrasonography during a routine medical checkup. She was subsequently referred to our hospital for investigation. Computed tomography (CT) of the chest, abdomen, and pelvis revealed a large tumor in IVC, right iliac and ovarian veins, which was derived from the uterus and extended into the right atrium and ventricle. We identified two extension pathways of intravenous tumor originated from the posterior uterine wall (Figure 1). Furthermore, the thrombus showed enhancement after administration of contrast material, which was indicative of tumor thrombus or benign metastasizing leiomyomatosis. On pelvic magnetic resonance imaging (MRI), an irregular tumor was identified in the right posterior wall of the uterus, which exhibited heterogeneous high signal intensity on T2-weighted images. Dynamic contrast-enhanced MRI using gadolinium with diethylenetriaminepentaacetate revealed the enhancement of the tumor in IVC (Figure 2). Because the lesion was located in the right atrium and ventricle, cardiovascular surgery consultation was recommended. A transthoracic echocardiogram was duly performed and revealed the tip of the tumor extended into the right atrium and also into the right ventricle. The multidisciplinary evaluation of the patient indicated that this was a case of cardiac-extending intravenous (IV) leiomyomatosis through the right ovarian and uterine veins arising from the uterine tumor.

**Figure 3:**
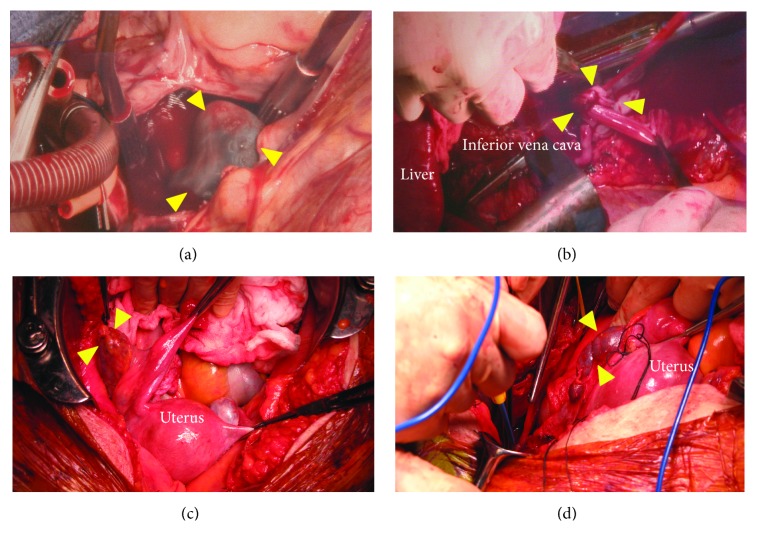
Intraoperative findings. Tumor in (a) the right atrium (arrow heads), (b) the inferior vena cava (arrow heads), (c) the right ovarian vein (arrow heads), and (d) the right uterine vein (arrow heads).

The operation was performed under general anesthesia (Figure 3). The heart and IVC were exposed by the right lateral thoracotomy and the midline incision of the abdomen. In case of the tumor adhesion to the hepatic and diaphragmatic IVC, visualization of these IVC enables removing the IV mass and repairing the veins safely. That is why the right thoracotomy approach was chosen. Cardiopulmonary bypass was initiated from the superior vena cava and the right femoral vein/IVC. Inflow was instituted from bilateral femoral arteries to the ascending aorta. A right atriotomy was performed, and a large elastic tumor was found occupying most of the right atrium and extending into the right ventricle and IVC. This intracardiac mass was free floating without invasion of the myocardium and was removed from the right atrium. Thereafter, the right ovarian vein was ligated at the IVC level, and longitudinal venotomies were made in the suprarenal and infrarenal vena cava. Then, the intra-IVC mass was removed easily, because the tumor was capsulated well and there was no adhesion to the IVC and the right atrium. A longitudinal venotomy was also performed in the right common iliac vein, and the tumor in the right internal iliac vein was excised. The common iliac vein and IVC were repaired by continuous sutures with prolene suture line.

**Figure 4:**
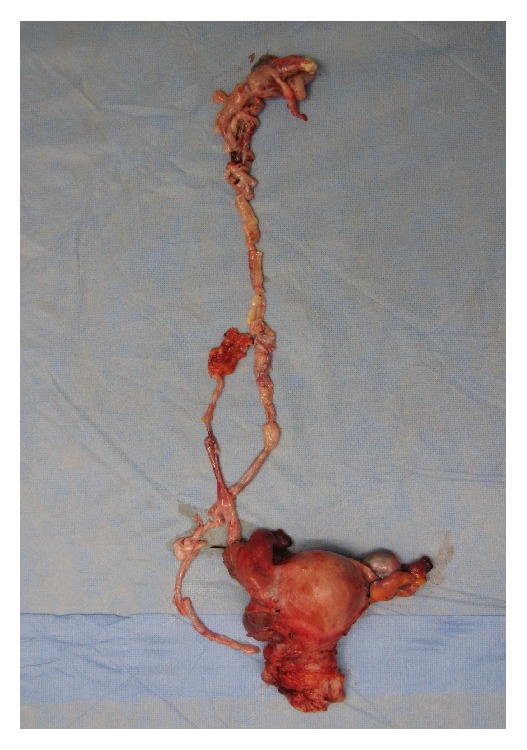
Extirpated uterus, bilateral ovaries and tubes, and tumor in the inferior vena cava extending from both the right uterine vein and common iliac and the right ovarian vein to the inferior vena cava and the right heart.

Next, the pelvis was explored. The uterus had enlarged to the size of a goose egg size where no myoma nodule was visible and tumors were found originating from the posterior wall of the uterus and continuing into both the right uterine and ovarian vein. The root of the tumor was attached to the lower posterior uterine serosa, and the stalks of the IV tumor were palpable within both veins continuing into the right internal iliac vein and IVC. The rest of the tumor was completely excised. Therefore, the patient underwent total hysterectomy with bilateral salpingooophorectomy. Complete tumor resection was achieved with an estimated blood loss of 1,045 mL (Figure 4).

**Figure 5:**
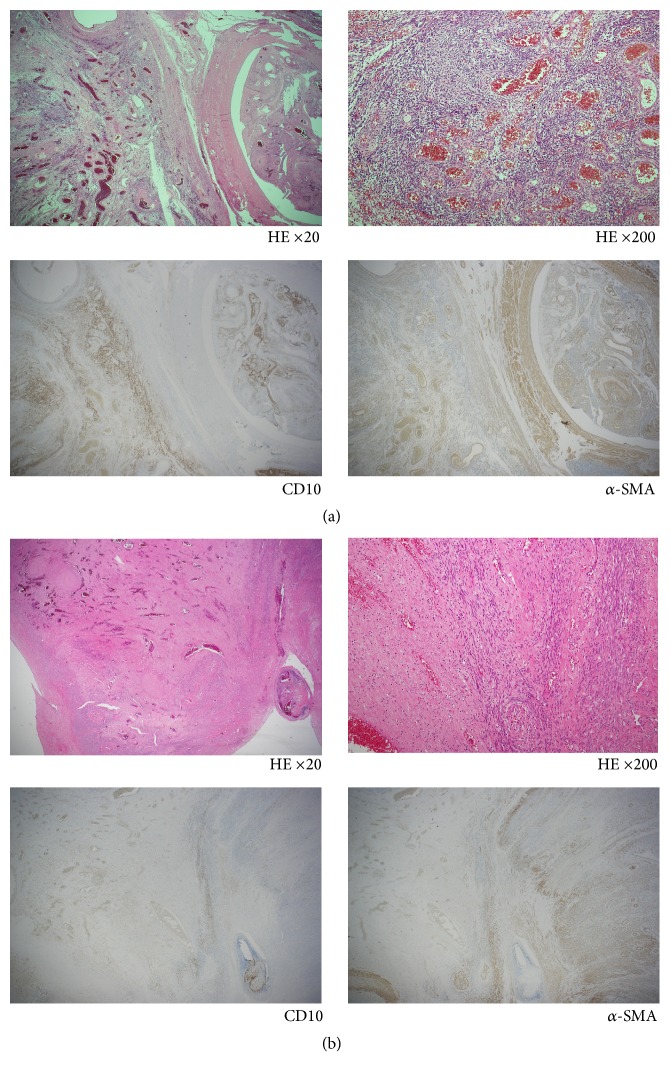
(a) Histopathological and immunohistochemical analyses show low-grade endometrial stromal sarcoma. The primary tumor in the uterus comprises CD10-positive short spindle cells resembling the stromal cells of proliferative endometrial tissue. Also spindle cells with abundant cytoplasm and ellipsoidal nuclei (*α*-SMA-positive cells) are mixed with the tumor cells. (b) The intracardiac tumor shows extensive smooth muscle differentiation. Main part is occupied with the spindle cells (*α*-SMA-positive). Scattered aggregates of small darker cells (CD10-positive) are still recognizable.

Histopathological analysis confirmed a diagnosis of LG-ESS. The primary tumor in the uterus comprised short spindle cells resembling the stromal cells of proliferative endometrial tissue infiltrating into the myometrium. These cells were positive for CD10. Also spindle cells with abundant cytoplasm and ellipsoidal nuclei are arranged in an interlacing bundle pattern (*α*-smooth muscle actin (SMA) positive cells) which were mixed with the tumor cells. Small vessels which are similar to spiral arteries and vessel invasion were noted. In contrast, the intracardiac tumor showed extensive smooth muscle differentiation. Main part was occupied with the spindle cells (*α*-SMA positive cells). The scattered aggregates of small darker cells (CD10 positive) as seen in prototypical LG-ESS were still recognizable. LG-ESS was histologically confirmed in the right parametria and IV tumor (Figure 5).

The patient experienced deep venous thrombosis in the right common iliac vein during her postoperative course. That was due to venous congestion induced by a stenosis of the repaired common iliac vein and was treated with heparin followed by warfarin. She received 600 mg/day medroxyprogesterone acetate (MPA) for 15 months as a postoperative adjuvant therapy and showed no evidence of disease during a follow-up period of 2 years and 3 months.

## 3. Discussion

This case was a rare example of LG-ESS that had extended to IVC and cardiac chambers and closely resembled IV leiomyomatosis. The clinical recognition of LG-ESS can be difficult and is often mistaken for a leiomyoma until a true diagnosis is made postoperatively, as was the case with our patient. The primary tumor in the uterus comprised CD10-positive short spindle cells, infiltrating into the myometrium. In contrast, the intracardiac tumor showed extensive smooth muscle differentiation, although the scattered aggregates of CD10-positive cells were still detectable. Immunophenotyping identified two distinct cell populations in the tumor and clearly illustrated the importance of extensive tissue sectioning and immunohistochemistry. CD10 protein expression is a relatively specific endometrial stromal marker in the uterus and can be used to differentiate endometrial stromal tumors from uterine smooth muscle tumors [8]. Mikami et al. also suggested that the predominance of a smooth muscle component in such a tumor can be misleading and does not always warrant a diagnosis of IV leiomyomatosis, nor does it predict a benign clinical course [9].

Preoperative planning should involve a multidisciplinary surgical team. In the present case, we used a multidisciplinary approach to evaluate our patient. Nogami et al. further highlighted that preoperative contrast-enhanced CT was unable to detect the free-floating intravascular tumors, thus illustrating a significant limitation of CT, and suggested that a more accurate determination of the extent of a specific tumor should be performed using multiple imaging methods when planning a surgical strategy [6]. Operative success is based on full anatomical exposure and complete vascular control, which is best achieved by full sternolaparotomy. Cardiopulmonary bypass provides excellent exposure and allows the safe removal of an intracardiac tumor thrombus [10]. Whenever possible, complete tumor clearance should be attempted. It has been shown that a free resection margin is the most important prognostic factor for long-term survival [11]. Our case highlights the importance of a multidisciplinary approach in treating this rare cardiovascular pathological condition. Accordingly, a radical resection with complete tumor removal was eventually possible.

Adjuvant hormonal therapy is an essential option for the prevention of recurrent ESS and for the treatment of residual, recurrent, or metastatic ESS following surgical resection [12, 13]. Because estrogen and progesterone receptors were detected in tissue samples from our patient, she received MPA for 15 months as postoperative adjuvant therapy. Hormonal therapy with progestins, aromatase inhibitors (third generation), and gonadotropin-releasing hormone analogues are also effective, and their use should be guided by the hormone receptor status of the tumor concerned [4]. Because of their growth-stimulating effects, both estrogen-based hormone replacement therapy and tamoxifen treatment are contraindicated [14, 15]. Because of the rarity of this type of tumor, no standardized or validated guidelines for adjuvant radio- and chemotherapy exist as yet.

In summary, the present case highlights the importance of a multidisciplinary approach in treating this rare cardiovascular pathological condition through preoperative assessment to final operation.
